# ATOM - an OMERO add-on for automated import of image data

**DOI:** 10.1186/1756-0500-4-382

**Published:** 2011-10-06

**Authors:** Oliver Müller, Peter Lipp, Lars Kaestner

**Affiliations:** 1 Institute for Molecular Cell Biology, Saarland University, Homburg, Germany; 2 Center for Bioinformatics Saar, Saarland University, Saarbrücken, Germany; 3 Research Center for Molecular Imaging and Screening, Saarland University, Homburg, Germany

## Abstract

**Background:**

Modern microscope platforms are able to generate multiple gigabytes of image data in a single experimental session. In a routine research laboratory workflow, these data are initially stored on the local acquisition computer from which files need to be transferred to the experimenter's (remote) image repository (e.g., DVDs, portable hard discs or server-based storage) because of limited local data storage. Although manual solutions for this migration, such as OMERO - a client-server software for visualising and managing large amounts of image data - exist, this import process may be a time-consuming and tedious task.

**Findings:**

We have developed ATOM, a Java-based and thus platform-independent add-on for OMERO enabling automated transfer of image data from a wide variety of acquisition software packages into OMERO. ATOM provides a graphical user interface and allows pre-organisation of experimental data for the transfer.

**Conclusions:**

ATOM is a convenient extension of the OMERO software system. An automated interface to OMERO will be a useful tool for scientists working with file formats supported by the Bio-Formats file format library, a platform-independent library for reading the most common file formats of microscope images.

## Background

The development of video enhanced microscope systems in the 1980s [1] has enabled digital microscope images in life sciences. Images were stored as a sequence of bits, analysed and organised with the aid of computer software. Initially, microscopes were predominantly operated manually, allowing a relatively low data throughput. With the emergence of high-content screening in the late 1990s [2] and thus the application of automated (i.e., software-controlled) microscopy platforms, image acquisition was accelerated [3]. As a consequence the number of stored digital images increased massively. These data had to be stored and organised following an imaging experiment. Moreover, an increasing variety of different image file formats emerged. To keep pace with this development, the imaging community requested a tool to handle arbitrary image file formats. In 2004, the Open Microscopy Environment (OME) consortium (University of Dundee, Scotland) developed the open-source software OME-Remote Objects (OMERO) and developed it further ever since [4,5]. OMERO allows users to visualise, manage and annotate digital microscope images and their corresponding metadata. Additionally, OMERO enables researchers and collaboration partners to share their digital image data over the intra- and internet. It provides the Java-based client OMERO.importer that can be used to read various image file formats and manually upload them to an OMERO server. However, manual import of image data has several drawbacks: (i) the user has to keep track and therefore spend time for the image file transfer; (ii) the transfer process cannot start until the file is closed. Manual import may thus lead to an increased booking time of the data acquisition (DAQ) system; (iii) if multiple images are recorded during an imaging experiment, manual import can either be done after each recording period or at the end of the experiment. While the first approach may lead to an interrupted workflow, the latter import compromises data safety.

## Approach

In the following sections we describe our software solution called AuTO.iMporter (ATOM), an OMERO add-on which enables users to circumvent most of the above mentioned shortcomings by automating the import process. Automation comprises monitoring of the DAQ system's image directory and periodic migration of new/modified image files to an OMERO server.

## Flow diagram of the approach

**Figure 1:**
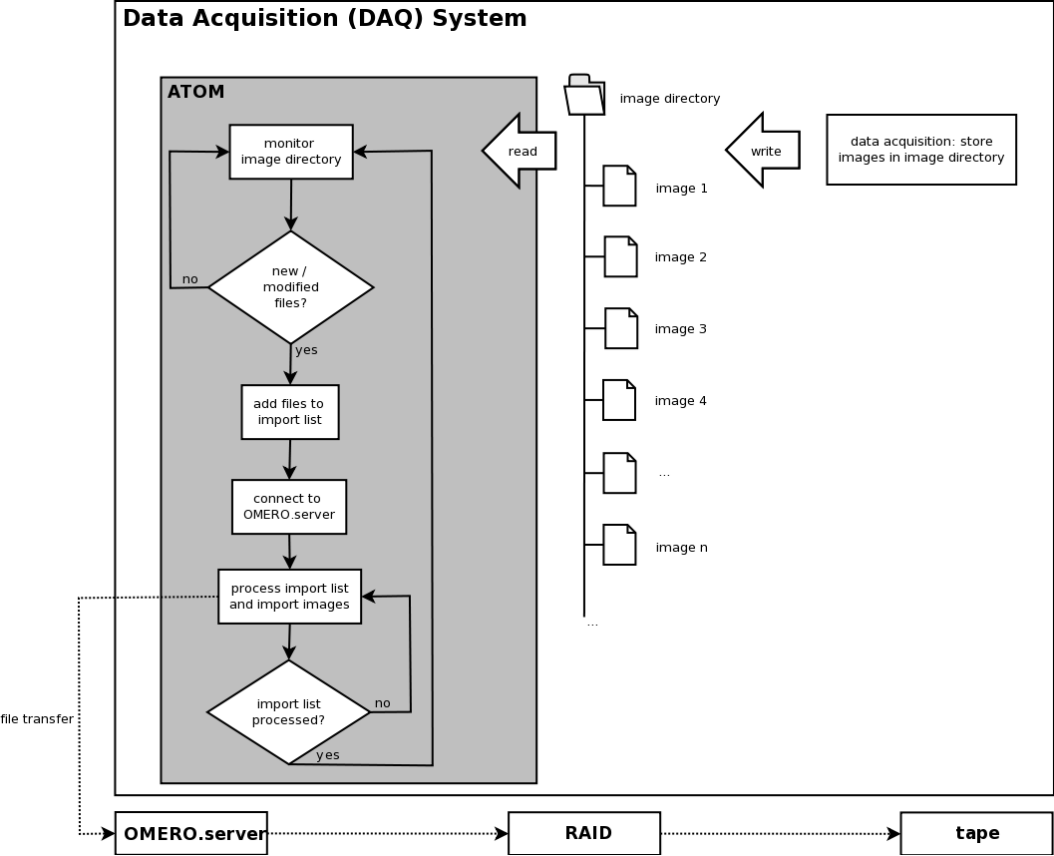
**Operation overview**. DAQ software stores image data in a defined image directory. Concurrently, ATOM monitors this directory and adds new/modified files to an import list. After connecting to the OMERO server software, ATOM processes the import list and migrates each file from the list to a remote OMERO server. Typically, images are then stored on a redundant array of independent discs (RAID) and additionally backed up on tape. Once the entire import list has been processed, ATOM continues monitoring the image directory.

The data flow of ATOM is depicted in Figure 1: during an imaging experiment, image files are stored in a local image directory on the DAQ system. This image directory has to be specified in ATOM and will be continuously monitored. In each monitoring cycle, new and modified files are migrated to the dedicated OMERO server automatically. Ideally, during an imaging experiment the transfer process should run in the background, without negatively affecting the DAQ process and the user interactions should be kept to a minimum. OMERO allows the user to organise image data following a hierarchical pattern: at the top level, the user may specify a project name. Projects again can contain multiple datasets. The latter typically contain the actual images. If the user has neither specified a project nor a dataset, images are imported "freely" and can be arranged according to the above mentioned hierarchy at a later time point. Following this strategy, ATOM allows the user to specify a predefined project/dataset. This enables the user to pre-organise images already during the imaging experiment. During each monitoring cycle, ATOM generates a snapshot of the image directory and stores its current state (i.e., filenames, creation and modification dates). The current snapshot is then compared to a previous one, enabling ATOM to detect new and modified files. Once all information about valid import candidates have been collected and stored in an import list, ATOM connects to the OMERO server and sequentially imports all files from the list. After the entire list has been processed, the connection to the server is closed again and the monitoring cycle restarts. Thereby, ATOM ensures a resource-saving operation. The cycle interval can be adapted by the user. A simple user's guide is provided as Additional File 1.

## Salient features

### Handling of multi-file formats

Automated import of image data implies rules for multi-file formats. These rules are defined in a dedicated Java class. While many file formats - like the commonly used Tagged Image File Format (TIFF) (Aldus Corporation, Seattle, USA) for instance - consist of a single image file that can be migrated to the OMERO server once it has been closed, other formats may be composed of multiple files. This is the case, if metadata and image data are stored separately. An example for such data handling is the VisiTech (Sunderland, United Kingdom) XYS file format. Here, image data are stored in a data file, while metadata are stored in a separate file. Both files are then linked using a third file. Importing this third file ensures that both, image data and metadata are imported correctly, while importing only one of the other files inevitably leads to an import of the incomplete dataset. In contrast, importing each of the three files leads to data duplication: both, image data and metadata then occur twice and occupy unnecessary storage space. To avoid such redundant data as well as to prevent separating and/or confusing metadata and image data, multi-file formats require special care. The OMERO.importer client per se has not been designed for automated import. Therefore, ATOM provides the functionality for handling multi-file image formats properly. In the case of the above mentioned XYS file format, the implemented rule causes ATOM to only import the file linking metadata and image data, if a file with suffix .xys has been found in the image directory. Since ATOM is open source, the above mentioned class can be updated and enhanced to support future multi-file formats.

### Graphical user interface

**Figure 2:**
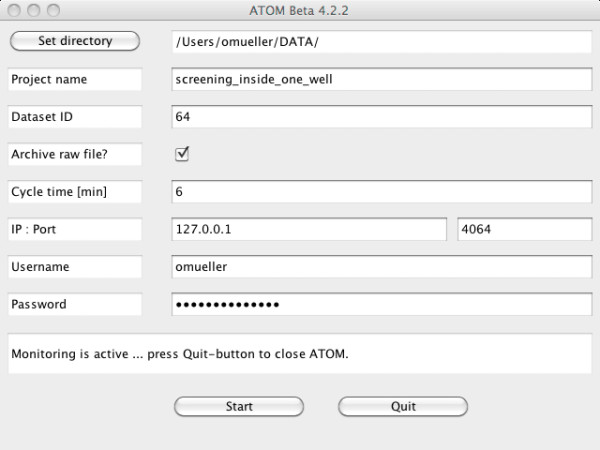
**Graphical user interface of ATOM**. Available for all major operating systems, the GUI allows to connect to an OMERO server and to monitor a specified image directory with a minimum amount of user interaction.

To make ATOM easy-to-use, it provides a graphical user interface (GUI), which is shown in Figure 2: the user has to specify an image directory, which is then monitored with a user-defined frequency. If no project name and dataset identifier are provided, images are imported using the default settings of OMERO. After specifying the server address and user account information, the monitoring process can be started. Messages describing the state of the import process are stored in a log file. The GUI is available for all major operating systems (see section "Availability and requirements" for details).

### ATOM in commercial high-content screening environments

During the last two years, an increasing number of vendors of automated microscope platforms such as Perkin Elmer ("Opera"), TILL Photonics ("more") or Leica Microsystems ("Leica HCS A") have recognised the benefits of OMERO. Having a quasi-standard for managing arbitrary image file formats enables cooperations between different research groups working with different DAQ systems. Therefore, vendors have started delivering their image acquisition platforms with an interface to OMERO, allowing users to store and share their image data in a common format. Thus, ATOM can serve as the interface to transfer image sequences in high-content/high-throughput applications.

## Implementation details

ATOM is implemented in Java (version 1.6.0) (Sun Microsystems, Santa Clara, USA) and thus provides platform-independence. It uses the application programming interface (API) of the OMERO.importer client (OME consortium, Dundee, Scotland) as well as the Java archive (JAR) file of the Bio-Formats file formats library (Laboratory for Optical and Computational Instrumentation, Wisconsin-Madison, USA). The latter is required for reading microscope image files and converting them into the OME file format. ATOM supports all DAQ platforms with file formats which can be handled by the Bio-Formats library. For a full list of compatible formats see [6].

For this publication, version 4.2.2 of the OMERO API has been used. Thus, ATOM is able to handle the same file formats as OMERO, i.e., all file formats supported by the Bio-Formats library [7]. To ensure compatibility between ATOM and the OMERO server, the ATOM version number must match the OMERO version number.

## Results

On our test site (Molecular Cell Biology, Homburg, Germany), four instances of ATOM were running simultaneously. Each set-up is equipped with a standard workstation (i.e., personal computers with an up to date quad-core processor and 4 GB of RAM). On average, each set-up generates 10 GB of image data (approximately 20 files) per imaging experiment. Using standard 100 Mbit/s ethernet cards, this results in a total transfer time of 15 minutes per set-up. Since the transfer is performed in the background during an experiment, this time does not add to the total booking time. To ensure data safety with respect to redundancy, in addition to the transfer of the data to the OMERO server, an additional copy of the data was also stored on the DAQ system. As a consequence, at this state of ATOM a manual delete process on the DAQ system was necessary after the data import into OMERO.

We have, for example, developed a high-content screening system for the analysis of primary cultured heart muscle cells incorporating an automated microscope platform [8]. In the meantime, this system has been enhanced by using OMERO as the image management system. About 15 GB of image data per experiment are automatically transferred to the OMERO server using ATOM. Since ATOM can be considered as an individual module, we believe that each screening environment can be easily enhanced to support automated image file transfer to an OMERO server.

## Discussion

### Comparison with OMERO system components

As an integral part of OMERO version beta 4 the OMERO.fs component (OME consortium, Dundee, Scotland), which provides the functionality of a file system monitor, has been released. Its first application OMERO.dropbox (OME consortium, Dundee, Scotland) pursues the same goal as ATOM but has a different approach: ATOM monitors a local image directory of the DAQ system while OMERO.dropbox monitors a remote subdirectory of the OMERO image repository. Thus, triggering an import process using OMERO.dropbox implies data transfer from the DAQ system to the remote directory. The development of OMERO.dropbox is still in progress. Currently, copying a large number of files "may result in files failing to import" [9]. Since monitoring a network-attached share (NAS) is "strictly not supported" [9], users have to manually copy their image data into the dropbox directory on the computer hosting the OMERO server, from where it is then imported into OMERO automatically. By circumventing such difficulties, ATOM provides a more convenient way for importing images into OMERO.

### ATOM in a multi-user environment

From the OMERO point of view, ATOM acts like any other OMERO client. Therefore it is obvious that multiple instances of ATOM running on dedicated set-ups can connect in parallel to one OMERO server. Nevertheless, this process as well as the login of each user into OMERO using the ATOM GUI (see Figure 2) can be further automated. A potential scenario could include software that is dedicated to run imaging core facilities, such as the Pasteur/Rockefeller Platform Management System (PPMS) [10]. This platform makes use of the user's PPMS login information to gain access to the DAQ system (to allow exact accounting). This could be further synchronised with the OMERO login information and thus even the start-up of ATOM could then be automated - either requesting the OMERO import information (project name & dataset ID) from the user or taking default values.

## Conclusions

ATOM is an easy-to-use add-on for OMERO, that offers automated import of digital images into an OMERO server, easing up data handling and increasing data safety significantly. Thus, ATOM is an interesting tool for scientists working with large amounts of imaging data in file formats that are supported by the Bio-Formats file format library.

## Availability and requirements

Project name: ATOM - AuTO.iMporter

Project home page: http://auto-importer.sourceforge.net/

Operating systems: Linux, MacOS X and Windows

Programming language: Java

Other requirements: Java 1.6.0 or higher, OMERO (note: the ATOM version number must match the OMERO version number)

License: GNU GPL

Restrictions to use by non-academics: none

## Abbreviations

API: Application programming interface; ATOM: AuTO.iMporter; DAQ: Data acquisition; DVD: Digital versatile disc; GB: Giga byte; GHz: Giga Hertz; ID: Identification; JAR: Java archive file; MBit: Mega bit; NAS: Network-attached share; OME: Open Microscopy Environment; OMERO: OME remote objects; RAID: Redundant array of independent discs; RAM: Random access memory; TIFF: Tagged Image File Format.

## Competing interests

The authors declare that they have no competing interests.

## Authors' contributions

OM has developed and programmed the software ATOM. OM, LK and PL have designed the research and equally contributed to the manuscript. All authors read and approved the final manuscript.

## Supplementary Material

Additional File 1 **User's guide**. A simple user's guide. Click here for file
