# Supplementary material for: ATOM - an OMERO add-on for automated import of image data
Source: BMC Res Notes. 2011 Oct 6;4:382. doi: 10.1186/1756-0500-4-382 (PMC3199262; doi:10.1186/1756-0500-4-382)
Supplement: Additional File 1 — User's guide. A simple user's guide. [file 1756-0500-4-382-S1.PDF]

Additional File 1
ATOM - an OMERO add-on for automated import of image data

Oliver Müller*[1,2], Peter Lipp[1,3] and Lars Kaestner[1,3]

[1]Institute for Molecular Cell Biology, Saarland University, Homburg, Germany
[2]Center for Bioinformatics Saar, Saarland University, Saarbrücken, Germany
[3]Research Center for Molecular Imaging and Screening, Saarland University, Homburg, Germany

Email: Oliver Müller*- oliver.mueller@bioinf.uni-sb.de; Peter Lipp - peter.lipp@uks.eu; Lars Kaestner - lars_kaestner@me.com;

*Corresponding author

User's guide
Download / installation

The software ATOM can be downloaded from http://auto-importer.sourceforge.net/. Since ATOM is implemented in Java, it is platform-independent and runs on all major operating systems. No installation is necessary. After downloading and extracting the software, it can be started as follows:

- *Apple Mac OS X*: execute the provided `.app` file
- *Linux*: execute the provided `.sh` file
- *Microsoft Windows*: execute the provided `.bat` file
- *Other Operating Systems*: you may try to execute the provided `.jar` file directly

The following sections describe how to set up ATOM using the GUI shown in Figure 2 of the article.

Organising image data transfer
Set directory

This button allows the user to select an arbitrary image directory to be monitored by ATOM. Usually this is the same directory used by the data acquisition software to store image data.

Project name

If a project name is entered, image data is imported into the specified project. Otherwise image data is imported "freely" and can be organised in OMERO at a later time point. This option may be used to

pre-organise image data.

Dataset ID

If a dataset ID is entered, image data is imported into the specified dataset. Otherwise image data is imported “freely” and can be organised in OMERO at a later time point. This option may be used to pre-organise image data.

Archive raw file

During an import process, image files are automatically converted to OMERO’s native file formats `OME-TIF` (image data) and `OME-XML` (metadata). The user may want to store the raw image files additionally, which can be achieved by checking the checkbox.

Cycle time

The user can enter a time interval n (in minutes) at which images are periodically migrated from the specified image directory to the OMERO server.

Entering server details

To import image data into OMERO, ATOM has to connect to a running OMERO server using pre-existing user information.

IP / Port

ATOM has to be provided with the valid IP address and port number of the OMERO server to be able to establish a connection for file import.

Username and password

Only users with an existing OMERO account are able to connect to the OMERO server and import their image data. This information has to be provided to ATOM.

Start

Pressing the start-button activates the monitoring process.

Quit

Pressing the quit-button exits ATOM.

Logfile

Information about the state of an import process are stored in a logfile in ATOM's `/log` directory.
